# Pyramidal Neurons Are Not Generalizable Building Blocks of Cortical Networks

**DOI:** 10.3389/fnana.2017.00011

**Published:** 2017-03-07

**Authors:** Jennifer I. Luebke

**Affiliations:** Department of Anatomy and Neurobiology, Boston University School of MedicineBoston, MA, USA

**Keywords:** mouse, rhesus monkey, comparative anatomy, dendrites, spines, synapses, visual cortex, prefrontal cortex

## Abstract

A key challenge in cortical neuroscience is to gain a comprehensive understanding of how pyramidal neuron heterogeneity across different areas and species underlies the functional specialization of individual neurons, networks, and areas. Comparative studies have been important in this endeavor, providing data relevant to the question of which of the many inherent properties of individual pyramidal neurons are necessary and sufficient for species-specific network and areal function. In this mini review, the importance of pyramidal neuron structural properties for signaling are outlined, followed by a summary of our recent work comparing the structural features of mouse (C57/BL6 strain) and rhesus monkey layer 3 (L3) pyramidal neurons in primary visual and frontal association cortices and their implications for neuronal and areal function. Based on these and other published data, L3 pyramidal neurons plausibly might be considered broadly “generalizable” from one area to another in the mouse neocortex due to their many similarities, but major differences in the properties of these neurons in diverse areas in the rhesus monkey neocortex rules this out in the primate. Further, fundamental differences in the dendritic topology of mouse and rhesus monkey pyramidal neurons highlight the implausibility of straightforward scaling and/or extrapolation from mouse to primate neurons and cortical networks.

The mammalian neocortex is a complex cellular structure endowed with exquisite computational powers. Excitatory pyramidal neurons and inhibitory interneurons together form long range and local networks which invest different cortical areas with the capacity to perform highly distinctive tasks such as visual encoding and mediation of executive functions. An influential idea has been that prototypical, generalizable pyramidal neurons comprise the basic building blocks of canonical cortical circuits or minicolumns which, once fully understood, can be extrapolated from one brain area or species to another (e.g., Douglas and Martin, 2004, 2007a,b). The view that individual neuron properties are conserved in different species and cortical areas has arisen from the existence of certain regularities in their basic design, the connections they have and the circuits they comprise, and it has been suggested that generalizable themes may allow for compression of connectomics data (in DeFelipe, 2015; DeFelipe et al., 2016).

The advent of large-scale brain mapping initiatives such as the Human Brain Project and the BRAIN Initiative highlight the need for ascertaining which, if any, data on the fundamental features of neurons can be extrapolated from one cortical area to another and from the rodent to the primate brain. In the attempt to understand an entity as vastly complex as the mammalian neocortex, simplification and a reductionist approach is, for the time being, unavoidable. The goal is to identify the smallest number of variables that will still allow for biologically realistic neuronal, network, and areal behavior in brain models; in other words, to be “simple but not too simple” (DeFelipe, 2015; DeFelipe et al., 2016). The degree of detail about individual neuron structure and function required for modeling species-specific cortical network functions is controversial (e.g., Kupferschmidt, 2015), though this depends on the complexity of population behaviors being modeled (reviews: Sporns, 2014; Yuste, 2015).

Much of what is known about pyramidal neuron structure and function has been derived from laboratory rat and mouse primary sensory cortices, and these data form the basis of large scale brain mapping initiatives (e.g., Human Brain Project, Allen Cell Types Database) and constrain realistic models directed toward elucidating cortical network mechanisms (Egger et al., 2014; Markram et al., 2015). Gaining a thorough understanding of similarities and differences in neurons in different cortical areas (e.g., primary sensory vs. association) in the mouse, and between the mouse and primates, is thus a high priority. Specifically, the degree to which mouse and primate neurons are similar or differ has profound relevance for the generalizability of brain maps from the mouse to the primate and for the degree to which information from mouse models of human brain disorders can or cannot be translated to non-human primates and ultimately to human beings.

## Structure-function relationships in pyramidal neurons- general themes

Cortical pyramidal neuronal processes are present in each of the 6 neocortical layers, with somata typically localized to layers 2–6 (except in layer 4c of the primary visual cortex, which is comprised exclusively of spiny stellate cells). The somata of pyramidal neurons are typically triangular, with a broad base from which a single axon and a skirt of basilar dendrites emanate and an apex from which, most typically, a single apical trunk projects. The apical dendrite has three compartments –a main trunk, oblique branches, a tuft that ramifies in layer 1- each of which possesses unique structural, connectional, and functional characteristics which broaden the dynamic range of signal integration by the apical dendritic arbor as a whole. The different dendritic compartments receive and integrate distinct presynaptic excitatory and inhibitory inputs and possess distinct passive and active signal filtering and boosting capacities (Larkum et al., 2001, 2009; London and Hausser, 2005; Losonczy and Magee, 2006; Losonczy et al., 2008; reviews: Spruston, 2008; Kubota et al., 2015, 2016). Differences in the lengths, diameters, and branching pattern of the dendritic arbor confer significant variability in cable properties and therefore the spatial distribution of electrical signals and degree of summation of synaptic inputs, which determine the temporal pattern of both forward and backward propagating action potentials (Mainen and Sejnowski, 1996; Koch and Segev, 2000; Euler and Denk, 2001; Vetter et al., 2001; Krichmar et al., 2002; Ascoli, 2003; reviews: Stuart et al., 1997; Waters et al., 2005). Thus, by virtue of their different somatodendritic compartments, pyramidal neurons act as coincidence detectors possessing a wide dynamic range for integration of temporally and spatially unique synaptic signals. Computational modeling studies suggest that even minor differences in branching characteristics can exert a major influence on signal processing by neurons. For example, even modest variations in branch point angles can transform the electrical coupling between oblique dendrites and the main apical shaft dendrite from fully coupled to fully compartmentalized (Ferrante et al., 2013).

Integration of synaptic inputs is also significantly shaped by active properties, including the number and distribution of a wide variety of transmembrane ion channels (reviews: Migliore and Shepherd, 2002; Magee and Johnston, 2005; Johnston and Narayanan, 2008). Over 20 different types of sodium, calcium, and potassium channels are distributed -some uniformly and some non-uniformly- across a given dendrite and confer distinct boosting and/or dampening of local signals (reviews: Migliore and Shepherd, 2002; Magee and Johnston, 2005; Johnston and Narayanan, 2008; Nusser, 2012). The complex interplay of intrinsic ionic and synaptic conductances with passive properties determined by dendritic morphology can effectively alter the cable properties of the dendritic tree (Segev and London, 2000; Bekkers and Häusser, 2007; review: Nusser, 2012) resulting in a variable and finely-tunable integrative and signaling capacity in pyramidal neurons.

Dendritic spines -principal recipients of glutamatergic synapses- also play a key role in the electrical and biochemical signaling in dendrites (reviews: Matus and Shepherd, 2000; Nimchinsky et al., 2002; Kasai et al., 2003; Bourne and Harris, 2008). While there is a continuum of spine morphology at steady state, and morphology can vary dynamically in response to synaptic activity (Lendvai et al., 2000; Zuo et al., 2005; for review: Lüscher et al., 2000; Wefelmeyer et al., 2016), spines can be broadly classified as being “thin,” “stubby,” “mushroom,” or “filopodia” (review: Bourne and Harris, 2008). Just as with dendrites, spine structural properties underlie functional physiological signaling; thus spine and synapse structure is largely determinative of the strength, stability and function of excitatory glutamatergic synapses (Tong and Jahr, 1994; Baude et al., 1995; Murthy et al., 1997, 2001; Nusser et al., 1998; Matsuzaki et al., 2001; Li et al., 2005; Germuska et al., 2006). Thus, quantification of the distribution of spine subtypes as well as of synapses on pyramidal neurons is essential for understanding the integrative capacities of these neurons in distinct brain areas and species.

## L3 pyramidal neuron morphology varies depending on brain area and species

Dendritic arbor size and spine density on pyramidal neurons differ markedly across functionally distinct cortical areas in the rhesus monkey and the human brain (Cajal SRy, 1894, 1995; Conel, 1941, 1967; DeFelipe et al., 2002; Jacobs, 2002; Elston, 2003; Elston and Fujita, 2014; Mohan et al., 2015). In the rhesus monkey, the size, and complexity of the dendritic arbors of L3 pyramidal neurons increases dramatically from primary visual cortex (V1) to higher-order lateral prefrontal cortex, and this increase in overall size is accompanied by a significantly higher spine density (Elston, 2000, 2002, 2003; Elston et al., 2001; Amatrudo et al., 2012). In addition to this caudal to rostral gradient observed in the primate, pyramidal neurons in some analogous cortical areas increase in size or “scale” from the rodent to the macaque (Elston and DeFelipe, 2002; Elston and Zeitsch, 2005; Ballesteros-Yanez et al., 2006; Elston, 2007; Wen et al., 2009; Elston and Manger, 2014; reviews: Wittenberg and Wang, 2008; DeFelipe, 2011).

To gain a deeper understanding of comparative morphological features of cortical neurons in rodents and primates, as well as their functional relevance, we have used *in vitro* whole-cell patch-clamp recordings and cell filling in a series of systematic studies to characterize the detailed structural, neuro-chemical and functional properties of L3 pyramidal neurons in the primary visual and frontal association cortices of mice and of rhesus monkeys (Amatrudo et al., 2012; Luebke et al., 2015; Medalla and Luebke, 2015; Gilman et al., 2016; Hsu et al., in press). A unique feature of these studies, which are summarized below, is that neurons were assessed both morphologically and physiologically at high resolution and in an identical manner across different brain areas in the two species allowing direct and comprehensive comparisons.

## L3 pyramidal neurons in primary visual and frontal association areas in the rhesus monkey and in the mouse

### Rhesus monkey

**Figure 1:**
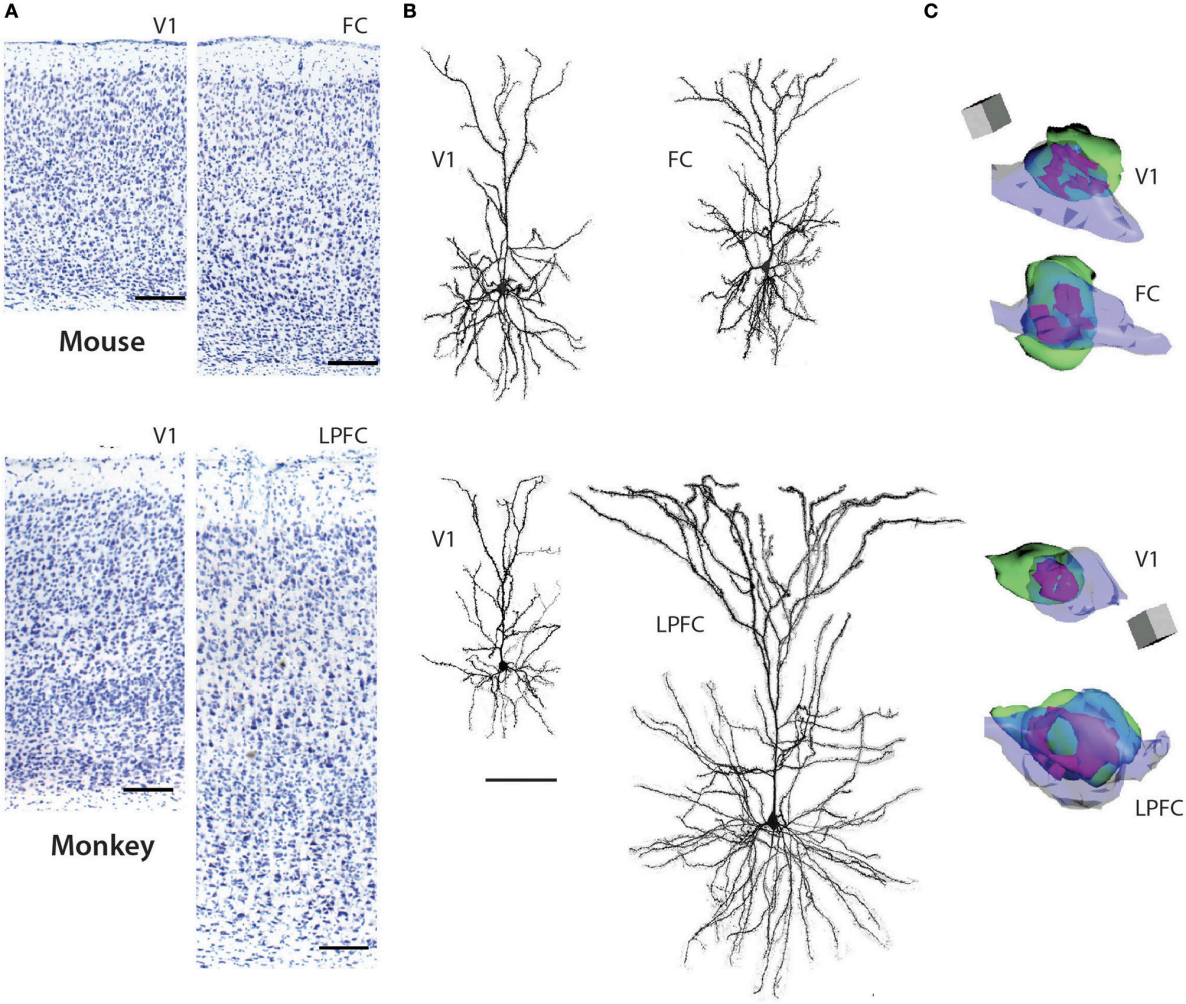
**Pyramidal neurons in V1 and FC (FR2) in the mouse (top panels) and V1 and LPFC of the rhesus monkey (bottom panels). (A)** 4x photomicrographs of Nissl-stained coronal sections of mouse V1, mouse FC, rhesus monkey V1, rhesus monkey LPFC. **(B)** Representative reconstructions of L3 pyramidal neurons (filled with biocytin during recordings and then processed with Alexa-streptavidin and imaged using confocal microscopy) from mouse V1, mouse FC, rhesus monkey V1, and rhesus monkey LPFC (ventral bank of the principal sulcus). Note the significantly larger size of the layer 3 pyramdial neuron from monkey LPFC compared to monkey V1 while these neurons do not differ in size in the mouse. **(C)** 3D reconstructions of representative axo-spinous perforted synapses in neuropil of mouse V1, mouse FC, rhesus monkey V1, and rhesus monkey LPFC. Spines are shown in green, boutons in blue, and perforated synapses in purple. Note the significantly larger perforated synapse as well as spine and bouton in monkey LPFC compared to monkey V1; these ultrastructural features do not differ in size in the two cortical areas in the mouse. Scale bars = A: 1 cm; B: 100 μm; C: 0.5 μm. **(A,B)** adapted from Gilman et al. (2016) and **(C)** from Hsu et al. (in press).

L3 pyramidal neurons in V1 and LPFC of the rhesus monkey are highly distinctive across a broad spectrum of structural and functional properties (Elston, 2000, 2002; Elston et al., 2001; Amatrudo et al., 2012; Zaitsev et al., 2012; Medalla and Luebke, 2015; Gilman et al., 2016; Hsu et al., in press). Most prominently, the dendritic arbors of LPFC neurons are on average 2.5x larger than those of V1 neurons and are also significantly more complex, with twice as many branch points (Figure 1; Table 1). The smaller size of V1 neurons is related to a higher input resistance, lower rheobase and higher evoked action potential firing rates compared to LPFC neurons (Amatrudo et al., 2012; Table 1). Further, the mean number and mean density of dendritic spines are ~5-fold and ~2-fold higher, respectively, on monkey LPFC than on V1 neurons (Elston and Rosa, 1997; Elston, 2003; Amatrudo et al., 2012; Medalla and Luebke, 2015; Table 1). Interestingly however the numeric density of asymmetric excitatory synapses in the layer 2/3 neuropil of these two areas does not differ (Hsu et al., in press). These apparently incongruous findings can be explained by the fact that the density of neurons in V1 (and hence the density of synapses) is significantly higher than in LPFC in the monkey. Electron microscopic assessment of excitatory synapse ultrastructure in layer 2/3 neuropil reveal that presynaptic boutons and postsynaptic densities of axospinous synapses are significantly larger in monkey LPFC compared to those in V1. It is of key functional significance that there is also a higher proportion of large perforated synapses in LPFC neuropil (Figure 1; Table 1) since this feature of postsynaptic densities is associated with long-term potentiation of glutamatergic synaptic responses (review: Lüscher et al., 2000; Wefelmeyer et al., 2016). The existence of larger synapses in LPFC, together with the much higher density of spines, likely provide the structural underpinning of the significantly larger and more frequent synaptic currents –that is, enhanced synaptic efficacy- seen in LPFC compared to V1 with whole-cell patch-clamp recordings (Amatrudo et al., 2012; Medalla and Luebke, 2015; Table 1).

**Table 1:** **Comparative structural and functional features of neurons and excitatory synapses in C57/BL6 mouse and rhesus monkey (***Macaca mulatta***) V1 and FC/LPFC**.

	**MouseV1**		**MouseFC**		**MonkeyV1**		**MonkeyLPFC**		**Mouse**	**Monkey**	**Mouse vs. Monkey**	**Mouse vs. Monkey**
	**Mean**	**SEM**	**Mean**	**SEM**	**Mean**	**SEM**	**Mean**	**SEM**	**Vl vs. FC**	**Vl vs. LPFC**	**Vl**	**FC and LPFC**
Soma Diameter (μm)	14.9	0.4	15.8	0.5	12.7	0.5	17.3	0.9	ns	*p* < 0.001	*p* < 0.001	ns
Dendritic Length (μm)	4,291	261	5,034	241	3,188	226	7,631	919	ns	*p* < 0.01	ns	*p* < 0.01
# Branch Points	44	3.5	49.3	2.9	32	2.6	51.3	5.6	ns	*p* < 0.01	ns	ns
Spine Number	4,377	302	4,819	534	1,884	216	10,018	2,062	ns	*p* < 0.001	*p* < 0.01	*p* < 0.01
Spine Density (sp/μm)	0.99	0.08	0.95	0.07	0.6	0.03	1.12	0.18	ns	*p* < 0.001	*p* < 0.05	ns
**Synapse Density (N**_v_ × **10^6^/mm^3^)**												
All Asym	0.93	0.08	1.05	0.13	0.48	0.06	0.45	0.04	ns	ns	*p* < 0.0001	*p* < 0.0001
Asym AxoSp	0.86	0.09	0.99	0.11	0.37	0.05	0.37	0.04	ns	ns	*p* < 0.0001	*p* < 0.0001
Asym AxoDen	0.07	0.02	0.06	0.02	0.12	0.01	0.08	0.00	ns	*p* < 0.043	*p* < 0.018	ns
% perforated synapses	23.3	0.4	19.5	4.0	20.2	4.7	34.8	1.5	ns	*p* < 0.003	ns	*p* < 0.004
**PSD area (**μ**m**^3^**)**												
All	0.084	0.008	0.075	0.020	0.082	0.010	0.116	0.009	ns	*p* < 0.028	ns	*p* < 0.017
Non-perforated	0.061	0.006	0.050	0.011	0.075	0.011	0.071	0.006	ns	ns	ns	ns
Perforated	0.152	0.021	0.160	0.030	0.113	0.013	0.199	O.018	ns	*p* < 0.003	ns	ns
**Spine volume (**μ**m**^3^**)**												
All	0.069	0.020	0.048	0.016	0.066	0.009	0.102	0.006	ns	*p* < 0.021	ns	*p* < 0.004
Non-perforated	0.050	0.012	0.034	0.010	0.061	0.008	0.061	0.005	ns	ns	ns	*p* < 0.025
Perforated	0.128	0.033	0.107	0.023	0.086	0.011	0.181	0.008	ns	*p* < 0.001	ns	*p* < 0.007
**Electrophysiology**												
Rn(MOhm)	229	12	215	17	224	21	102	9	ns	*p* < 0.001	ns	*p* < 0.001
Rheobase (pA)	96.7	7.6	81.9	7	80.2	8.3	144.7	15.8	ns	*p* < 0.05	ns	*p* < 0.05
80 pA FR (APs/sec)	5.6	0.7	5.4	1.3	14.9	1.8	5.4	1.8	ns	*p* < 0.001	*p* < 0.001	ns
sEPSC Freq (Hz)	4.6	0.4	3.3	0.2	1.2	0.2	2.9	0.5	ns	*p* < 0.05	*p* < 0.01	*p* < 0.01
sEPSC Amp (pA)	13.5	1.5	9.8	0.5	7.3	0.4	14	2.1	ns	*p* < 0.01	ns	ns
sEPSC Rise (ms)	1.75	0.11	1.61	0.07	1.22	0.1	1.85	0.21	ns	*p* < 0.01	ns	ns
sEPSC Decay (ms)	7.7	0.34	6.62	0.18	4.63	0.5	7.77	0.69	ns	*p* < 0.01	ns	ns

*Light microscopy level morphometric data on somata, dendrites, and spines and electrophysiology data are compiled from Amatrudo et al. (2012), Luebke et al. (2015), Medalla and Luebke (2015) and Gilman et al. (2016). Electron microscopy level data on synapse density and size are compiled from Medalla and Luebke (2015) and Hsu et al. (in press). Numbers of subjects and cells analyzed for each variable are provided in these original citations*.

### Mouse

Whether, there is a homolog for the primate LPFC in the mouse or rat has been a matter of some discussion and debate (Preuss, 1995; Uylings et al., 2003; Kolb, 2007; Wise, 2008; Van De Werd et al., 2010; Barbas, 2015). The mouse cortical area that is arguably the closest anatomical analog is the dorsomedial frontal cortex (including area FR2) which receives dense inputs from the mediodorsal nucleus of the thalamus (Guldin et al., 1981; Van De Werd et al., 2010) just as the LPFC of the primate does. In marked contrast to the significant differences in L3 pyramidal neurons observed between these two areas in the rhesus monkey, L3 pyramidal neurons in the mouse V1 and FC (FR2) exhibit very modest differences in dendritic structural properties -V1 neurons being slightly smaller than FC- and are nearly identical with regard to physiological features assessed *in vitro* (Table 1). In the mouse there is also no areal difference in the number or density of dendritic spines on V1 and FC L3 pyramidal neurons or in the ultrastructural properties of excitatory synapses in the two areas. Predictably, excitatory synaptic currents are similarly indistinguishable between mouse FC and V1 neurons by marked contrast to the major differences in these currents between monkey LPFC and V1 (Table 1).

## Structure of neurons in primary visual and frontal association areas in the mouse compared to the rhesus monkey

While frontal cortical L3 pyramidal neurons scale significantly in size from the mouse to the monkey, no such scaling exists with L3 pyramidal neurons in V1 (Gilman et al., 2016). That dendritic scaling occurs in frontal but not visual L3 pyramidal neurons provides interesting insight into potentially differential capabilities of these neurons in the two species. As frontal cortical pyramidal neurons increase in size across phylogeny, the opportunity for convergence of diverse inputs is increased, as is their integrative and computational dynamic range. As discussed above, integration and filtering of input signals occurs as a function of number of dendritic branch points and both diameter and the geometric features –notably length and diameter- of dendritic segments (Rall, 1962, 1964). Scaling and cable theory predict that monkey LPFC neurons filter input signals to a greater extent than mouse FC neurons due to their greater dendritic length and equivalent dendritic diameters. Consistent with this, there is a higher frequency of spontaneous EPSCs in mouse FC vs. monkey LPFC (Gilman et al., 2016). On the other hand, action potential firing and other intrinsic properties are largely preserved between mouse and monkey frontal neurons, suggesting significant roles for non-passive properties (e.g., active conductances and synaptic inputs; Nusser, 2012) that should be examined in future studies. V1 neurons in the monkey possess the lowest number and density of spines while spine densities do not significantly differ between monkey LPFC and mouse FC and mouse V1 neurons, though given their much smaller size, the mean numbers of spines on pyramidal neurons in the mouse is much lower than in monkey LPFC (Gilman et al., 2016). The ultrastructural properties of excitatory synapses vary across species in that the L2-3 neuropil of monkey LPFC contains a significantly higher proportion of perforated postsynaptic densities and, on average, larger spine volume compared to L2-3 neuropil of mouse FC. Finally, both presynaptic and postsynaptic entities are significantly smaller in the mouse FC than in the monkey LPFC.

## Implications for species-specific cortical areal specialization

L3 pyramidal neurons in mouse FC and V1 are virtually identical in their dendritic, spine, and excitatory synapse structure as well as in their physiological properties. These findings are in line with the high degree of cytoarchitectural and functional homogeneity across mouse cortical areas, compared to the highly specialized cortical areas of the primate brain. The similarity in the structural and biophysical properties of mouse V1 and FC neurons suggests that relatively similar temporal signaling dynamics may exist within these areas. In mice, both the primary sensory area V1 and the multimodal FC contain cellular and synaptic features consistent with a highly excitable circuit, being comprised of small and electrically compact output neurons, and abundant spines with relatively small excitatory synapses. Thus, in broad terms mouse cortical neurons (and presumably the dynamic networks of which they are a part) are well suited for rapid synaptic transmission with a high degree of input-output fidelity but relatively low dynamic range (review: Olshausen and Field, 2004; Vogels et al., 2005; Panzeri et al., 2010).

In contrast to the small and much less differentiated rodent neocortex, a larger and more specialized brain, such as that of the rhesus monkey, requires functionally distinct cortical areas to have different levels of excitability, filtering, and integration of inputs (Luebke et al., 2010; Barbas, 2015). The preponderance of morphological and electrophysiological data predict that in the monkey, synaptic integration at the cellular and network levels differ between V1, a primary sensory area for unimodal representation, and LPFC, a high-order area for complex multimodal processing (review: Schummers et al., 2004; Fuster, 2015). V1 neurons are small, compact and highly excitable, properties that enable them to respond optimally to small, fast synaptic inputs and for building a network with a limited dynamic range but well-suited for signal transformations with relatively high input-output fidelity (review: Olshausen and Field, 2004; Vogels et al., 2005; Panzeri et al., 2010). Compared to the primary sensory V1, the multimodal association LPFC in monkey is comprised of cellular and synaptic features—large and electrotonically complex L3 output neurons with many spines- consistent with more powerful and longer-lasting inputs. These features are optimal for facilitating sustained activation, coincidence detection, and spike-timing-dependent plasticity, all important for integrative functions such as decision making and integration of sensorimotor information (review: Constantinidis and Wang, 2004; Sjostrom et al., 2008). The function of the LPFC is to integrate multimodal information from a wide array of cortical and subcortical afferents in order to perform sophisticated executive tasks (review: Miller and Cohen, 2001; Luebke et al., 2010). A relatively larger dynamic range of integration of information conferred by larger neurons with more numerous synapses is required in a high-order area such as LPFC, while it would be disadvantageous in V1 where more rapid signal transformations are required.

## Conclusions

In terms of their fundamental structural properties -dendrite, spine, and synapse morphology- there are some striking and many subtle differences between L3 pyramidal neurons in the mouse and the rhesus monkey and between cortical areas in the rhesus monkey but not in the mouse. In the mouse, where L3 pyramidal neurons are structurally the same in these two brain areas, a uniform prototypical cortical pyramidal neuron may be generalizable from one area to another, at least in terms of size, dendritic structure, and intrinsic membrane, synaptic, and action potential firing properties. In the rhesus monkey such a prototypical neuron does not exist- cortical areas differ markedly from each other at the individual pyramidal cell and network levels. Data such as those summarized here are important for understanding how signaling within neuronal networks differs between rodents and primates and for how these neurons and networks may contribute to species-specific functional capacities. These findings however do not answer the question of *which*, if any, of these particular differences are necessary and sufficient for differentiating neuronal network behavior in different brain areas and species. The answer to this question remains to be determined and is difficult to predict, particularly in light of *in silico* predictions that neuronal networks display emergent behavior that may not depend on details of individual neuron structure and function (review: Yuste, 2015). For now, since we do not know which of myriad details about individual neurons are key for network function, the tendency for premature simplification should be avoided (DeFelipe et al., 2016). Our understanding of neuronal diversity in all of its complexity is nascent, but thanks to advances in molecular, genetic and neuroanatomical tools we are on the verge of a new era in which the great diversity of neuronal types will be cataloged and lead to more nuanced and comprehensive insights into the mechanisms of cortical areal specialization.

## Author contributions

The author confirms being the sole contributor of this work and approved it for publication.

### Conflict of interest statement

The author declares that the research was conducted in the absence of any commercial or financial relationships that could be construed as a potential conflict of interest. The reviewer MR and handling Editor declared their shared affiliation, and the handling Editor states that the process nevertheless met the standards of a fair and objective review.

## Abbreviations

sp: spine
asym: asymmetric (excitatory) synapse
PSD: postsynaptic density
Rn: input resistance
FR: firing rate
Aps: action potentials
sEPSC: spontaneous excitatory synaptic current
freq: frequency
amp-: amplitude.
